# Revolutionizing Small Molecule Drug Discovery Pipelines with Cryo-EM: A Workflow for High-Throughput Screening and High-Resolution Data Collection

**DOI:** 10.1063/4.0001138

**Published:** 2025-10-27

**Authors:** Adrian F Koh, Victoria Cushing, Basil Greber, Pascal Lill, Surajit Banerjee, Zuben Brown, Abhay Kotecha

**Affiliations:** 1 Thermo Fisher Scientific, Eindhoven, North Brabant, Netherlands; 2 The Institute of Cancer Research, Sutton, London, United Kingdom; 3 Thermo Fisher Scientific, Portland, Oregon, USA

## Abstract

Cryo-electron microscopy (cryo-EM) has revolutionized structure-based drug discovery by allowing accurate and rapid visualization of drug-target interactions at high resolutions1. In this study, we demonstrate a workflow using a 200 kV Thermo Scientific Glacios cryo-transmission electron microscope (cryo-TEM) equipped with a Thermo Scientific Selectris X imaging filter and Thermo Scientific Falcon 4i detector for high-throughput screening of drug binding on CDK-Activating Kinase (CAK) complex. With just one hour of data collection, we obtained structures at 4 Å, sufficient to identify lead compound binding pocket and density. With four hours of data collection, the structures approached 3 Å, enabling visualization of lead compound conformation. Best grids imaged using the 300 kV Thermo Scientific Krios cryo-TEM resolved the structure to 2 Å, allowing the modelling of ordered waters to inform lead compound optimization. Our study demonstrates that 200 kV cryo-TEMs can deliver the high productivity needed for structure-based drug discovery and design, while targeted imaging on 300 kV cryo-TEMs can provide even higher resolutions.

